# Prevalence and Risk Factors of Left Ventricular Diastolic Dysfunction in Patients With Hyperthyroidism

**DOI:** 10.3389/fendo.2020.605712

**Published:** 2021-01-08

**Authors:** Huan Li, Renli Zeng, Yunfei Liao, Mengfei Fu, Huan Zhang, Linfang Wang, Yuming Li

**Affiliations:** ^1^ Department of Endocrinology, Union Hospital, Tongji Medical College, Huazhong University of Science and Technology, Wuhan, China; ^2^ Hubei Provincial Clinical Research Center for Diabetes and Metabolic Disorders, Wuhan, China; ^3^ Department of Gastrointestinal Surgery, Union Hospital, Tongji Medical College, Huazhong University of Science and Technology, Wuhan, China

**Keywords:** left ventricular diastolic dysfunction, hyperthyroidism, prevalence, risk factors, echocardiography

## Abstract

**Background:**

Left ventricular (LV) diastolic dysfunction has been demonstrated to be an independent predictor of the future heart failure. Heart failure is one of the severe complications caused by overt hyperthyroidism. However, the effects of overt hyperthyroidism on diastolic dysfunction are conflicting, and little is known about the prevalence and risk factors of the diastolic dysfunction in patients with overt hyperthyroidism.

**Methods:**

A total of 388 patients with overt hyperthyroidism were included and compared with 388 age- and gender- matched euthyroid control subjects. LV diastolic function was evaluated by traditional and tissue-Doppler echocardiography. Routine clinical medical data and echocardiographic parameters were recorded for analysis.

**Results:**

The prevalence of LV diastolic dysfunction was 35.1% among hyperthyroid patients and significantly higher than control subjects whose prevalence was 25.5% (*P* = 0.003), and it increased with age and body mass index (BMI) in patients with overt hyperthyroidism. The possible risk factors for LV diastolic dysfunction, such as hypertension, diabetes, decreased estimated glomerular filtration rate (eGFR), and increased level of thyroid hormones weren’t associated with LV diastolic dysfunction. However, overweight or obese were significantly associated with LV diastolic dysfunction (OR = 3.024, 95% CI = 1.517–6.027, *P* = 0.002) compared with normal BMI. When compared with age <40 years old group, 40-50 years old group, 50-60 years old group and age ≥60 years old group were significantly associated with LV diastolic dysfunction, with ORs of 2.976 (95% CI = 1.744–5.019), 12.424 (95% CI = 4.934–31.283), 24.966 (95% CI = 5.975–104.321), respectively.

**Conclusion:**

LV diastolic dysfunction was very common, in particular, in older and overweight or obese patients with overt hyperthyroidism. Additionally, age and BMI were independent risk factors for LV diastolic dysfunction, while the level of thyroid hormones was not. Therefore, besides the LV systolic function, we also need focus on the diastolic function in patients with overt hyperthyroidism in clinical work, especially the older and overweight or obese patients.

## Introduction

Hyperthyroidism is one of the most common endocrine diseases, affecting 0.2%–1.3% of the people in iodine-sufficient places of the world (1), which constitutes a major health issue due to its prominent association with cardiovascular diseases, including congestive heart failure, atrial fibrillation, and pulmonary hypertension and so on (2, 3).

Studies showed that approximately 6% of patients with overt hyperthyroidism developed congestive heart failure (4), but only 50% of them had left ventricular (LV) systolic dysfunction. Besides LV systolic dysfunction, LV diastolic dysfunction has been demonstrated to be an independent predictor of the future heart failure (5–7). Therefore, LV diastolic dysfunction may account for part of reasons for heart failure in patients with overt hyperthyroidism. A meta-analysis indicated that the median prevalence of LV diastolic dysfunction was 36.0% (range 15.8%–52.8%) in older adults, and the results from the China Hypertension Survey reported that 35.5% of subjects existed LV diastolic dysfunction among Chinese adults aged ≥35 years (8, 9). However, there are few data on the prevalence of LV diastolic dysfunction in patients with overt hyperthyroidism.

Risk factors for LV diastolic dysfunction have been widely studied in general population, including body mass index (BMI), diabetes, hypertension, subclinical hyperthyroidism, etc (8, 10–12), but limited data are available on the risk factors for diastolic dysfunction among patients with overt hyperthyroidism. Furthermore, the results of studies on the effects of thyroid hormones on LV diastolic function are contradictary. To our best knowledge, some studies showed that patients with overt hyperthyroidism had an enhanced LV diastolic function (13, 14). However, there are not a few studies showing that overt hyperthyroidism is associated with LV diastolic dysfunction (15–18). Moreover, the sample of all those studies is relatively small.

Accordingly, this study aimed to investigate the prevalence and risk factors of LV diastolic dysfunction among patients with overt hyperthyroidism.

## Materials and Methods

### Subjects

A total of 388 patients receiving radioiodine treatment for overt hyperthyroidism were included in our study between May 2018 and May 2019 in the inpatient department of endocrinology, Union Hospital, Tongji Medical College, Huazhong University of Science and Technology, Wuhan, China. All included subjects were newly diagnosed patients with overt hyperthyroidism or had withdrawn anti-thyroid drug for at least 4 weeks. As defined in the guidelines of the American Thyroid Association Guidelines, overt hyperthyroidism was defined as increased free thyroxine (FT4) and/or free triiodothyronine (FT3) level and a concomitantly suppressed thyroid-stimulating hormone (TSH) level (19). In addition, we included 388 age- and gender- matched euthyroid control subjects who were free of current or past thyroid dysfunction and history of thyroid surgery. The euthyroid controls were included from the Medical Examination Center where healthy subjects underwent health check up regularly, and the transthoracic echocardiography and thyroid function test were just one part of their examinations. The exclusion criteria were as follows: 1) age <18 years; 2) with congenital heart disease or a history of cardiac surgery or other previous known heart diseases; 3) with atrial fibrillation or atrial flutter; 4) with acute or chronic renal failure; 5) with malignancy. All enrolled subjects signed the informed consent agreement and approval was obtained from the Ethics Committees of Tongji Medical College of Huazhong University of Science and Technology.

### Data Collection

Data were collected on the demographic characteristics, physical examinations, history of diseases and laboratory examinations, including age, sex, height, weight, heart rate (HR), systolic blood pressure(SBP), diastolic blood pressure (DBP), duration of symptoms of hyperthyroidism, as well as levels of serum uric acid (SUA), creatinine (Scr), electrolytes (including sodium, potassium, and calcium), TSH, FT4, FT3, anti-thyroglobulin antibody (TgAb), anti-thyroid peroxidase antibody (TPOAb), and thyrotropin receptor antibody (TRAb). BMI was calculated as the weight (kg) divided by height (m) squared. According to the recommended criteria for the Chinese population, BMI was categorized into underweight (<18.5 kg/m2), normal (18.5–24.0 kg/m2), overweight (24.0–28.0 kg/m2), and obese (28.0 kg/m2) (20). Overweight and obese were grouped together for analysis, because the sample size of subjects with obese is relatively small. The estimated glomerular filtration rate (eGFR) was calculated on the basis of Scr by using the Chronic Kidney Disease Epidemiology Collaboration equation (CKD-EPI) (21).

Diabetes mellitus (DM) was defined as fasting blood glucose ≥7.0 mmol/L or postprandial blood glucose ≥11.1 at least twice during the period in inpatient, or any self-reported history of DM or the use of anti-diabetic medications. Hypertension was defined as SBP ≥140 mmHg or (and) DBP ≥90 mmHg at least twice in inpatient, or any self-reported history of hypertension or the use of oral anti-hypertensive medications. Hyperuricemia was defined as SUA concentration >420 mmol/L in male and >360 mmol/L in female.

### Transthoracic Echocardiography

All the enrolled subjects underwent complete transthoracic echocardiography using echocardiography system with a 3~8 MHz transducer (GE Vivid 7; Vingmed; Philips EPIQ 7C and Philips IE33). The definition of LV diastolic dysfunction were performed by two experienced sound operator according to the mitral E/A ratio or (and) the septal basal regions e/a ratio. The cut off was less than 1, which indicates impaired myocardial relaxation (22). We recorded the left ventricular end-diastolic diameter (LVEDD), interventricular septum thickness (IVST), left atrium diameter (LAD), and left ventricular ejection fraction (LVEF). In addition, the peak velocities of the early (E-wave) (MVE) and late (A-wave) (MVA) phase of the mitral inflow pattern were measured from an apical four-chamber view.

### Statistical Analysis

Whether the continuous variables conformed to normal distribution was assessed by using the Kolmogorov-Smirnov test. The normally distributed data were expressed as mean ± standard deviation (SD), while the skewed distribution data were expressed as median (interquartile range). All categorical data were presented as numbers or percentages as indicated. The paired t test was used for the normally distributed matched continuous data. Comparisons between non-normally distributed matched continuous data were made by suing the Wilcoxon signed rank test. McNemar test was used for the matched nominal data. For the independent variables analysis, the intergroup differences were analyzed by the Student’s t-test, the Mann-Whitney test, or the Chi-square test, as appropriate. In the hyperthyroid group, spearman’s correlation analysis was undertaken to explore the associations between LV diastolic function parameters and other clinical parameters; stepwise models of multiple linear regression analysis were performed to explore the impact of independent variables on LV diastolic function parameters; multivariable logistic regression analysis was conducted to identify independent factors associated with LV diastolic dysfunction. *P*-values < 0.05 were considered to be statistically significant. All statistical analyses were performed with SPSS 22.0 (SPSS, Chicago, Illinois, USA).

## Results

### Characteristics of the Hyperthyroid and Control Subjects

The characteristics of the overt hyperthyroidism patients and control subjects are shown in **Table 1**. A total of 388 patients diagnosed with overt hyperthyroidism and 388 age- and gender- matched euthyroid control subjects were included in this study according to the inclusion and exclusion criteria. The median age was 38 years old (interquartile range, 29–48) for both group (*P* = 0.782), and 67.3% of them were female. The median serum FT3 and FT4 of hyperthyroid subjects were 25.6 pmol/l and 61.4 pmol/l, respectively; the serum TSH was undetectable in 284 patients (TSH < 0.005). The serum FT4 and TSH levels of all control subjects were at normal range. Compared with the control subjects, the hyperthyroid subjects tended to have higher SBP (*P* < 0.001), higher HR (*P* < 0.001), higher level of eGFR (*P* < 0.001), as well as lower BMI (*P* < 0.001), lower DBP (*P* = 0.001), and lower level of Scr (*P* < 0.001). However, there was no significant difference in the incidences of hyperuricemia, hypertension and DM between these two groups.

**Table 1:** Comparison of characteristics between the hyperthyroid and normal control subjects.

	Control N = 388	Overt hyperthyroidism N = 388	*P*-value
Age (years)	38 (29–48)	38 (29–48)	0.782
Female, n(%)	261 (67.3%)	261 (67.3%)	1.000
BMI (kg/m2)	22.6 (20.6–24.6)	20.8 (19.1–22.7)	<0.001
SBP (mmHg)	120 (115–130)	128 (117–137)	<0.001
DBP (mmHg)	79 (74–85)	77 (70–84)	0.001
HR (bpm)	78 (70–81)	93 (81–105)	<0.001
FT3 (pmol/L)	4.6 (4.2–5.0)	25.6 (16.2–36.0)	<0.001
FT4 (pmol/L)	12.7 (11.9–13.7)	61.4 (39.9–95.5)	<0.001
Undetectable thyrotropin	0	284 (73.2%)	<0.001
SUA (umol/L)	295 (239–365)	310 (259–363)	0.094
Scr (mg/dl)	0.67 (0.58–0.79)	0.49 (0.40–0.61)	<0.001
eGFR (ml/min/1.73m2)	112.2 ± 14.8	127.4 ± 16.6	<0.001
Comorbidities			
Hyperuricemia	72 (18.6%)	68 (17.5%)	0.769
Hypertension	26 (6.7%)	19 (4.9%)	0.310
DM	10 (2.6%)	21 (5.4%)	0.072
Echocardiographic evaluation			
LV diastolic dysfunction	99 (25.5%)	136 (35.1%)	0.003
LAD (cm)	3.1 (2.9–3.4)	3.2 (3.0–3.5)	<0.001
LVEDD (cm)	4.5 (4.2–4.7)	4.5 (4.3–4.8)	0.002
IVST (cm)	0.9 (0.8–1.0)	0.9 (0.8–1.0)	0.048
LVEF (%)	65 (63–68)	65 (63–68)	0.549
MVE (m/s)	0.8 (0.7–1.0)	1.0 (0.8–1.2)	<0.001
MVA (m/s)	0.7 (0.6–0.8)	0.8 (0.7–1.0)	<0.001
E/A ratio	1.3 (1.1–1.5)	1.3 (1.0–1.5)	0.193

Data are presented as number (%), mean (SD), or median (interquartile range) according to the distribution. BMI, body mass index; SBP, systolic blood pressure; DBP, diastolic blood pressure; HR, heart rate; FT3, free triiodothyronine; FT4, free thyroxine; SUA, serum uric acid; Scr, serum creatinine; eGFR, estimated glomerular filtration rate; DM, diabetes mellitus; LV, Left ventricular; LAD, left atrium diameter; LVEDD, left ventricular end-diastolic diameter; IVST, interventricular septum thickness; LVEF, left ventricular ejection fraction; MVE, peak velocities of the early (E-wave) phase of the mitral inflow pattern; MVA, peak velocities of the late (A-wave) phase of the mitral inflow pattern; E/A ratio, the ratio of MVE/MVA.

In the comparisons of echocardiographic examination, the prevalence of LV diastolic dysfunction was 35.1% (136 patients) among those patients with hyperthyroidism, whereas it was 25.5% (99 subjects) in the control group (*P* = 0.003). Furthermore, the LAD, LVEDD, IVST, MVE, and MVA were significantly higher in hyperthyroid subjects compared with the control subjects (all *P* < 0.05).

### Basic and Echocardiographic Characteristics of the Hyperthyroid Subjects With or Without LV Diastolic Dysfunction

We compared the demographic and clinical characteristics between hyperthyroid subjects with or without LV diastolic dysfunction. The results are shown in **Table 2**. Compared with patients with normal LV diastolic function, the subjects with LV diastolic dysfunction tended to be older (*P* < 0.001), and have higher BMI (*P* < 0.001), higher level of serum sodium (*P* = 0.002), lower eGFR (*P* < 0.001), and lower level of serum FT4 (*P* = 0.012).In addition, the incidences of hypertension and DM were also higher in patients with diastolic dysfunction compared with those with normal LV diastolic function (*P* < 0.05). Although the serum level of FT3 tended to be lower in subjects with diastolic dysfunction, the difference was not significant.

**Table 2:** Basic characteristics of the hyperthyroid subjects with or without LV diastolic dysfunction.

	Without diastolic dysfunction N = 252	With diastolic dysfunction N = 136	*P*-value
Age (years)	33 (26–43)	48 (39–55)	<0.001
Duration (months)	12.0 (2.0–60.0)	24.0 (2.0–84.0)	0.226
Female, n(%)	168 (66.7%)	93 (68.4%)	0.731
BMI (kg/m^2^)	20.2 (18.8–22.0)	21.7 (19.7–23.6)	<0.001
BMI (n)			0.001
Underweight	50	14	
Normal	177	93	
Overweight or obese	25	29	
SBP (mmHg)	126 ± 15	128 ± 17	0.364
DBP (mmHg)	77 ± 11	76 ± 11	0.502
HR (bpm)	95 (81–105)	92 (81–102)	0.205
Comorbidities			
Hyperuricemia	43 (17.1%)	25 (18.4)	0.744
Hypertension	8 (3.2%)	11 (8.1%)	0.032
DM	9 (3.6%)	12 (8.8%)	0.029
SUA (umol/L)	311 (260–362)	310 (249–371)	0.654
Scr (mg/dl)	0.49 (0.39–0.60)	0.48 (0.42–0.61)	0.519
eGFR (ml/min/1.73m^2^)	131.5 ± 15.6	119.8 ± 15.8	<0.001
Sodium (mmol/L)	141 (139–142)	142 (140–143)	0.002
Potassium (mmol/L)	3.95 (3.75–4.17)	3.94 (3.74–4.14)	0.742
Calcium (mmol/L)	2.30 ± 0.12	2.30 ± 0.14	0.970
FT3 (pmol/L)	27.0 (17.7–36.9)	22.0 (14.9–34.6)	0.055
FT4 (pmol/L)	65.1 (41.9–100)	52.9 (38.7–86.4)	0.012
TSH (mIU/L)			0.19
<0.005	179 (71%)	105 (77.2%)	
≥0.005	73 (29.0%)	31 (22.8%)	
TPOAb (IU/ml)	262.7 (75.3–389.6)	280.1 (89.9–549.8)	0.344
TgAb (IU/ml)	127.3 (19.0–539.7)	203.3 (17.6–539.7)	0.533
TRAb (IU/L)	13.8 (7.1–23.7)	11.2 (5.0–21.1)	0.122

Data are presented as number (%), mean (SD), or median (interquartile range) according to the distribution. LV, Left ventricular; BMI, body mass index; SBP, systolic blood pressure; DBP, diastolic blood pressure; HR, heart rate; DM, diabetes mellitus; SUA, serum uric acid; Scr, serum creatinine; eGFR, estimated glomerular filtration rate; FT3, free triiodothyronine; FT4, free thyroxine; TSH, thyroid-stimulating hormone; TPOAb, anti-thyroid peroxidase antibody; TgAb, anti-thyroglobulin antibody; TRAb, thyrotropin receptor antibody.

In the comparisons of echocardiographic parameters, LAD, IVST and MVA were significantly higher in patients with diastolic dysfunction (both *P* < 0.05). MVE and E/A ratio were significantly lower in subjects with diastolic dysfunction compared to those with normal diastolic function (both *P* < 0.001). The results are presented in **Table 3**.

**Table 3:** Echocardiographic parameters of the hyperthyroid subjects with or without LV diastolic dysfunction.

Echocardiographic parameters	All N = 388	Without diastolic dysfunction N = 252	With diastolic dysfunction N = 136	*P*-value
LAD (cm)	3.2 (3.0–3.5)	3.2 (3.0–3.5)	3.3 (3.0–3.7)	0.043
LVEDD (cm)	4.5 (4.3–4.8)	4.5 (4.3–4.8)	4.5 (4.3–4.8)	0.576
IVST (cm)	0.9 (0.8–1.0)	0.9 (0.8–0.9)	0.9 (0.9–1.0)	<0.001
LVEF (%)	65 (63–68)	65 (63–68)	65 (63–69)	0.193
MVE (m/s)	1.0 (0.8–1.2)	1.0 (0.9–1.2)	0.9 (0.7–1.0)	<0.001
MVA (m/s)	0.8 (0.7–1.0)	0.8 (0.6–0.9)	0.9 (0.8–1.0)	<0.001
E/A ratio	1.3 (1.0–1.5)	1.3 (1.2–1.6)	1.0 (0.8–1.0)	<0.001

Data are presented as median (interquartile range). LV, Left ventricular; LAD, left atrium diameter; LVEDD, left ventricular end-diastolic diameter; IVST, interventricular septum thickness; LVEF, left ventricular ejection fraction; MVE, peak velocities of the early (E-wave) phase of the mitral inflow pattern; MVA, peak velocities of the late (A-wave) phase of the mitral inflow pattern; E/A ratio, the ratio of MVE/MVA.

### Associations Between LV Diastolic Function Parameters and Other Clinical Parameters in Hyperthyroid Subjects

The correlations between LV diastolic function parameters and other clinical parameters in patients with overt hyperthyroidism are shown in **Table 4**. The age, BMI, levels of serum sodium and Scr were negatively correlated with MVE, while the female, eGFR, serum levels of FT3 and FT4, as well as LAD were positively correlated with MVE. MVA was negatively correlated with serum levels of Scr and TSH, but positively correlated with age, female, BMI, SBP, HR, the presence of hypertension, LAD, and IVST, as well as serum levels of FT3 and FT4. As for E/A ratio, it was negatively correlated with age, BMI, serum level of sodium, the presence of hypertension, LAD, and IVST, and positively correlated with eGFR and serum level of TSH.

**Table 4:** The correlations between LV diastolic function parameters and other clinical parameters in hyperthyroid subjects.

	MVE		MVA		E/A ratio	
	Rs	*P* -value	Rs	*P* -value	Rs	*P* -value
Age	−0.193	<0.001	0.209	<0.001	−0.355	<0.001
Female	0.160	0.002	0.175	0.001	−0.050	0.330
BMI	−0.178	<0.001	0.105	0.038	−0.250	<0.001
SBP	0.047	0.356	0.141	0.005	−0.075	0.139
DBP	0.025	0.624	0.076	0.134	−0.052	0.305
HR	0.068	0.184	0.180	<0.001	−0.098	0.054
eGFR	0.253	<0.001	−0.060	0.241	0.267	<0.001
SUA	−0.058	0.254	−0.010	0.845	−0.033	0.511
Scr	−0.233	<0.001	−0.209	<0.001	0.018	0.727
Sodium	−0.104	0.042	0.055	0.283	−0.148	0.004
Potassium	0.032	0.530	−0.065	0.204	0.085	0.093
Calcium	−0.001	0.980	0.074	0.145	−0.067	0.185
Hyperuricemia	−0.065	0.200	0.026	0.605	−0.083	0.103
Hypertension	−0.014	0.790	0.143	0.005	−0.145	0.004
DM	−0.033	0.516	0.056	0.271	−0.077	0.186
FT3	0.143	0.005	0.195	<0.001	−0.080	0.115
FT4	0.179	<0.001	0.201	<0.001	−0.052	0.308
TSH	−0.039	0.447	−0.155	0.002	0.122	0.016
LAD	0.125	0.014	0.233	<0.001	−0.124	0.014
LVEDD	0.021	0.680	−0.004	0.942	0.037	0.471
IVST	−0.085	0.095	0.131	0.010	−0.205	<0.001
LVEF	0.091	0.073	0.087	0.088	−0.009	0.856

LV, Left ventricular; BMI, body mass index; SBP, systolic blood pressure; DBP, diastolic blood pressure; HR, heart rate; eGFR, estimated glomerular filtration rate; SUA, serum uric acid; Scr, serum creatinine; DM, diabetes mellitus; FT3, free triiodothyronine; FT4, free thyroxine; TSH, thyroid-stimulating hormone; LAD, left atrium diameter; LVEDD, left ventricular end-diastolic diameter; IVST, interventricular septum thickness; LVEF, left ventricular ejection fraction.

Based on the correlation analysis, multiple linear stepwise regression analysis was performed to identify the related factors that were critical determinants of the three LV diastolic function parameters. As shown in **Table 5**, age was the only independent variable associated with the MVE, MVA, and E/A ratio. Additionally, Sex, BMI, eGFR, and LAD were independently associated with MVE. The HR, SBP, LAD, as well as serum level of FT3 and Scr were independently associated with MVA. HR, BMI, and serum level of FT3 were independently associated with E/A ratio.

**Table 5:** Potential factors related to the parameters of LV diastolic function in hyperthyroid subjects.

	MVE			MVA			E/A ratio		
	B (SE)	β	*P*	B (SE)	β	*P*	B (SE)	β	*P*
Age	−0.003 (0.001)	−0.150	0.049	0.004 (0.001)	0.244	<0.001	−0.012 (0.001)	−0.414	<0.001
Female	0.080 (0.024)	0.161	0.001						
HR				0.002 (0.001)	0.183	<0.001	−0.004 (0.001)	−0.171	0.001
SBP				0.002 (0.001)	0.120	0.011			
BMI	−0.017 (0.004)	−0.195	<0.001				−0.029 (0.006)	−0.225	<0.001
eGFR	0.002 (0.001)	0.158	0.035						
Scr				−0.222 (0.073)	−0.152	0.003			
FT3				0.002 (0.001)	0.114	0.034	−0.004 (0.001)	−0.160	0.001
LAD	0.157 (0.025)	0.321	<0.001	0.076 (0.021)	0.173	<0.001			

Age, gender, HR (heart rate), SBP (systolic blood pressure), BMI (body mass index), eGFR (estimated glomerular filtration rate), the presence of hypertension, the level of FT3 (free triiodothyronine), FT4 (free triiodothyronine), TSH (thyroid-stimulating hormone), and Scr (serum creatinine), as well as LA (left atrium diameter) and IVST (interventricular septum thickness) were included in the multiple stepwise linear regression model. LV, Left ventricular; MVE, peak velocities of the early (E-wave) phase of the mitral inflow pattern; MVA, peak velocities of the late (A-wave) phase of the mitral inflow pattern; E/A ratio, the ratio of MVE/MVA.

### The Risk Factors for LV Diastolic Dysfunction in Hyperthyroid Subjects

We analyzed the relationships between various parameters and the presence of LV diastolic dysfunction on the basis of the univariate analysis. The results were presented in **Table 6**. The univariable logistic regression analysis showed that age, BMI, the presence of hypertension or DM, eGFR, and serum level of FT4 were significantly related to LV diastolic dysfunction. In the multivariable logistic regression analysis, only age and BMI were significantly associated with LV diastolic dysfunction. Compared with normal BMI, the overweight or obese was significantly associated with LV diastolic dysfunction (OR = 3.024, 95% CI = 1.517–6.027, *P* = 0.002), while the underweight was not (*P* > 0.05). Compared with age <40 years old group, the 40–50 years old group, 50–60 years old group and age ≥60 years old group were significantly associated with LV diastolic dysfunction, with ORs of 2.976 (95% CI = 1.744–5.019), 12.424 (95% CI = 4.934–31.283), 24.966 (95% CI = 5.975–104.321), respectively.

**Table 6:** Univariable and multivariable logistic regression analyses for independent risk factors of LV diastolic dysfunction in hyperthyroid subjects.

Variables	Univariate		Multivariate	
	OR (95% CI)	*P*-value	OR (95% CI)	*P*-value
Age				
<40	1.000 (Ref)		1.000 (Ref)	
40–50	2.958 (1.744–5.019)	<0.001	2.976 (1.744–5.019)	0.002
50–60	10.905 (5.525–21.522)	<0.001	12.424 (4.934–31.283)	<0.001
≥60	20.132 (6.444–62.888)	<0.001	24.966 (5.975–104.321)	<0.001
BMI				
Underweight	0.533 (0.280–1.014)	0.055	0.431 (0.199–0.933)	0.033
Normal	1.000 (Ref)		1.000 (Ref)	
Overweight/obese	2.208 (1.223–3.986)	0.009	3.024 (1.517–6.027)	0.002
Hypertension	2.684 (1.053–6.843)	0.039	0.630 (0.194–2.041)	0.441
DM	2.613 (1.072–6.369)	0.035	1.234 (0.415–3.673)	0.705
eGFR	0.951 (0.936–0.966)	<0.001	0.997 (0.972–1.022)	0.796
FT3	0.985 (0.969–1.002)	0.078	1.030 (0.998–1.064)	0.067
FT4	0.990 (0.982–0.997)	0.008	0.987 (0.973–1.002)	0.100
TSH	0.724 (0.446–1.175)	0.191		

LV, Left ventricular. Multivariable logistic regression model included age, BMI (body mass index), the presence of hypertension or DM (diabetes mellitus), eGFR (estimated glomerular filtration rate), as well as the level of FT3 (free triiodothyronine) and FT4 (free triiodothyronine).

### The Prevalence of LV Diastolic Dysfunction Grouped by Age or BMI in Hyperthyroid Subjects

**Figure 1:**
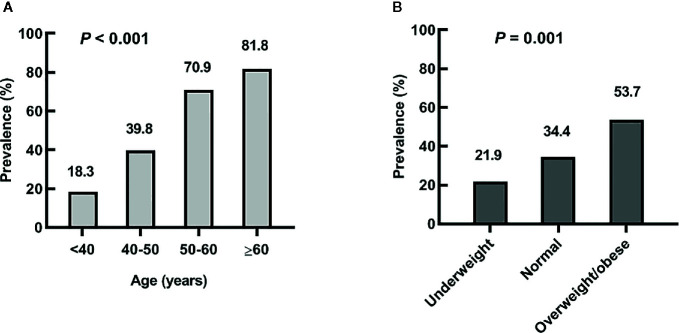
The prevalence of left ventricular diastolic dysfunction in overt hyperthyroidism patients grouped by age or body mass index (BMI). **(A)** The subjects were grouped by age; **(B)** The subjects were grouped by BMI categories.

We further explored the prevalence of LV diastolic dysfunction in different age or BMI groups. The results are showed in **Figure 1**. The prevalence of diastolic dysfunction in patients with overt hyperthyroidism increased with age and BMI. 18.3% of patients in <40 years old group, 39.8% of patients in 40–50 years old group, 70.9% of patients in 50–60 years old group, and 81.8% of patients in >60 years old group had LV diastolic dysfunction (*P* < 0.001). For different BMI groups, 21.9% of patients in underweight group, 34.4% of patients in normal group, and 53.7% of patients in overweight/obese group had LV diastolic dysfunction (*P* = 0.001).

## Discussion

In present study, we investigated the prevalence and risk factors of LV diastolic dysfunction among patients with overt hyperthyroidism enrolled in the inpatient department of endocrinology of our hospital. We found that the overall prevalence of LV diastolic dysfunction was 35.1% in patients with overt hyperthyroidism, close to the reported prevalence in older adults from a meta analysis and the results from the China Hypertension Survey, which was 36%, 35.5% respectively (8, 9). Additionally, compared with the age- and gender- matched euthyroid control group, the prevalence was significantly higher in hyperthyroid group. When the age or BMI was categorized, the prevalence of LV diastolic dysfunction in hyperthyroid subjects increased with age and BMI. Additionally, age and BMI were positively associated with the presence of LV diastolic dysfunction in patients with overt hyperthyroidism, independent of other possible risk factors, including the presence of hypertension or DM, eGFR, as well as serum level of FT3 and FT4.

Heart failure was one of the severe complications caused by overt hyperthyroidism, but only 50% of patients with overt hyperthyroidism had reduced LV ejection fraction (4, 16). It was well acknowledged that LV diastolic dysfunction was an independent predictor of future heart failure, so we inferred that LV diastolic dysfunction may account for part of the incidences of heart failure in patients with overt hyperthyroidism. However, there are no established effective treatments for LV diastolic dysfunction. Therefore, it is important to know the current condition and risk factors of LV diastolic dysfunction. Although there were many studies on the prevalence of LV diastolic dysfunction, few were performed in patients with overt hyperthyroidism. A study from Yue showed that the prevalence of diastolic dysfunction was 31% in patients with overt hyperthyroidism, and the prevalence increased with age: 17.9% in patients <40 years to 100% in those >60 years (16). Nevertheless, the included subjects in this study were much smaller (only 70 patients), and they didn’t explore the effect of BMI and other comorbidities on the prevalence of diastolic dysfunction. Furthermore, they didn’t include an age- and gender- matched normal control group for analysis. Our data may more accurately represent the condition of diastolic dysfunction in patients with overt hyperthyroidism.

urthermore, univariable and multivariable logistic analysis were performed to find all possible factors that might related to LV diastolic dysfunction in our study. Age and BMI were defined as the independent risk factors, which was consistent with results of other kinds of populations (10, 23). A work from Hamza et al. found that deterioration of LV diastolic function was striking age-related and started at a young adult age (23). It is generally accepted that age is an independent risk factors of cardiovascular diseases. With aging, continuous cardiomyocytes stress, like the increased formation of reactive oxygen species and imbalances of mitochondrial fusion/fission, can injure the cardiomyocytes, while the cardiomyocytes’ regeneration is limited, which lead to cardiac structure changes and electrophysiological dysfunction ultimately (24, 25). Furthermore, previous studies reported that the vascular stiffness, left ventricle wall thickness, and fibrosis increased with age (26), which can contribute to the LV diastolic dysfunction. As for BMI, previous studies demonstrated that a higher BMI was associated with worse LV diastolic function, and weight loss could improve diastolic function for obese subjects (10, 27). Our present study first reported that BMI was also associated with LV diastolic function in patients with overt hyperthyroidism. The mechanisms may be that the higher BMI can lead to increased preload, afterload and peripheral resistance (28). Besides, it has been demonstrated that increased adiposity enhances the adverse effect of blood pressure on LV mass growth (29). A higher BMI usually indicate higher adiposity, and hyperthyroidism always contributes to hypertension. Eventually, the increased LV mass would lead to impaired myocardial relaxation.

Previous studies have reported that the hypertension, DM and renal function were associated with the impaired LV diastolic function (8, 11, 30). Although the univariable logistic analysis showed that the hypertension, DM and eGFR were associated with LV diastolic dysfunction, this associations disappeared in multivariable logistic analysis. We implied that the reason might be that the incidences of hypertension or DM in our enrolled subjects were relatively low and the renal function of them was all in normal range in present study. When the confounding factors were controlled, this association would not exist.

We also found that the serum level of FT3 independently correlated with MVA, E/A ratio, but the serum level of FT4 and TSH did not correlated with LV diastolic parameters in the stepwise linear regression models, which were in accordance with the results of Aroditis et al. (17). However, the work of Aroditis didn’t explore the correlation between the serum level of FT3 and LV diastolic parameters, and the confounding variables were not controlled. Although FT3 significantly correlated with some of the LV diastolic parameters, our study didn’t define the serum level of FT3 as an independent risk factor in multivariable logistic analysis. It is well known that FT3 is the primary biologically active form of thyroid hormone and has many effects on cardiovascular. Increased FT3 level could activate the synthesis of some cardiac structure protein, and lead to increased LV mass which is associated with impaired myocardial relaxation. However, previous studies confirmed that FT3 can downregulate the expression of phospholamban and upregulate sarcoplasmic the expression of reticulum calcium-activated ATPase, which results in increased rate of calcium reuptake, and enhanced cardiac relaxation (31, 32). We speculated that LV diastolic function may partially depend on the balance of increased LV mass and increased calcium reuptake. Thereby, FT3 may be a protective factor for some patients, while risk factor for others, such as the older and the overweight or obese patients.

Our study had some limitations. First, the assessment of LV diastolic dysfunction was relatively simple. We didn’t measure E’ velocity, E/E’ ratio and isovolumetric relaxation time etc which were associated with LV diastolic function, and we didn’t grade the diastolic dysfunction. Second, the sample was relatively small. Finally, although all subjects have withdrawn anti-thyroid drug, we didn’t explore the effect of other kinds of drugs on LV diastolic function, like the use of beta-blocker.

In conclusion, we found that LV diastolic dysfunction was more common in overtly hyperthyroid patients than the age- and gender- matched euthyroid control subjects, and the prevalence of LV diastolic dysfunction increased with age and BMI. Additionally, age and BMI were independent risk factors for the presence of LV diastolic dysfunction in patients with overt hyperthyroidism, while the serum levels of thyroid hormones were not. So we need to focus more on heart diastolic function of hyperthyroidism patients who are older and overweight or obese in our clinical work.

## Data Availability Statement

The data that support the findings of this study are available from the corresponding author on reasonable request. Requests to access these datasets should be directed to YL, lym.1020@163.com.

## Ethics Statement

The studies involving human participants were reviewed and approved by the Committees of Tongji Medical College of Huazhong University of Science and Technology. The patients/participants provided their written informed consent to participate in this study.

## Author Contributions

YML and LW conceived the study. YML, LW, and HL developed the study design and methods. HL and RZ collected the data. HL conducted data analysis and wrote the manuscript. HL, YFL, RZ, MF, and HZ researched the data. All authors contributed to the article and approved the submitted version.

## Funding

This study was supported by the Natural Science Foundation of Hubei province (2013CFB091) from the Science and Technology Department of Hubei.

## Conflict of Interest

The authors declare that the research was conducted in the absence of any commercial or financial relationships that could be construed as a potential conflict of interest.
